# Supplementary material for: Predicting the risk of asthma attacks in children, adolescents and adults: protocol for a machine learning algorithm derived from a primary care-based retrospective cohort
Source: BMJ Open. 2020 Jul 23;10(7):e036099. doi: 10.1136/bmjopen-2019-036099 (PMC7380838; doi:10.1136/bmjopen-2019-036099)
Supplement: Supplementary data [file bmjopen-2019-036099supp001.pdf]

ONLINE SUPPLEMENT 1: READ CODES LISTS USED TO DEFINE AN ASTHMA EVENT, ASTHMA MEDICATION AND POTENTIAL PREDICTORS

Asthma Diagnosis

"H33.. ", "H330. ", "H3300", "H3301", "H330z", "H331. ", "H3310", "H3311", "H331z", "H332. ", "H333. ", "H334. ", "H33z. ", "H33z0", "H33z1", "H33z2", "H33zz".

Asthma Medications

SABA Codes:

"x01Cq", "x01Cr", "c14r.", "c141.", "c14a.", "c14y.", "c149.", "c14c.", "c14z.", "c142.", "c14b.", "x006b", "c14t.", "c144.", "c14u.", "c145.", "c14v.", "c146.", "c14j.", "c14f.", "c14w.", "c147.", "c14x.", "c14i.", "c14g.", "x01Cs", "c14s.", "c143.", "c14h.", "c14e.", "c14..", "c14k.", "c148.", "x00Af", "x05Fg", "c11x.", "c111.", "c113.", "c115.", "c11e.", "c118.", "c11v.", "c112.", "c114.", "c116.", "c11y.", "c11k.", "c11a.", "c11d.", "c11n.", "c11p.", "c11o.", "c11q.", "c11w.", "c117.", "c11z.", "c11m.", "c11D.", "c11g.", "c11h.", "c11b.", "x05Fi", "c13v.", "c13a.", "c131.", "c132.", "c13p.", "c13H.", "c133.", "c13h.", "c13i.", "c134.", "c13J.", "c13I.", "c13Z.", "c13Y.", "c13T.", "c13R.", "c1E7.", "c13N.", "c13P.", "c13G.", "x00CE", "c13c.", "c131.", "c13n.", "c13K.", "c13O.", "c13U.", "c13V.", "c1E1.", "c13S.", "c13Q.", "c13L.", "c1E8.", "c1E2.", "c13M.", "c13x.", "c13q.", "c136.", "c13y.", "c13r.", "c137.", "c13C.", "x025g", "c13d.", "c1E3.", "c13D.", "x025h", "c13e.", "c1E4.", "c13E.", "x025i", "c13f.", "c1E5.", "c13F.", "x025j", "c13g.", "c1E6.", "c13w.", "c13W.", "c13A.", "c135.", "c13o.", "c13X.", "c13B.", "c13m.", "c13z.", "c139.", "c13j.", "x05Fh", "c12x.", "c124.", "c121.", "c12y.", "c125.", "c122.", "c12z.", "c126.", "c123.", "c12w.", "c1E9.", "c1EA.", "c1EB.", "c1EC.", "c1ED.", "c1EE.", "c11..", "c11A.", "c11B.", "c11C.", "c13..", "c138.", "c13b.", "c13s.", "c12..", "c1E..", "c51H.", "c51B.", "c51x.", "x0100", "x00zz", "c51v.", "c51w.", "c51C.", "c51D.", "c51F.", "c51E.", "c531.", "c51A.", "c51i."

LAB codes:

"c1C..", "c1C1.", "c1C2.", "c1C3.", "c1C4.", "c1C5.", "c1C6.", "c1C7.", "c1C8.", "c1Cy.", "c1Cz.", "c19..", "c191.", "c192.", "c193.", "c194.", "c195.", "c196.", "c197.", "c198.", "c199.", "c19A.", "c19B.", "c19z.", "c1b..", "c1b1.", "c1b2.", "c1b3.", "c1b4.", "c1d..", "c1d1.", "c1d2."

SAMA codes:

"x01Db", "c31..", "c31x.", "c311.", "c31z.", "c313.", "c31t.", "c31G.", "c31u.", "c315.", "c31A.", "c318.", "c31B.", "c319.", "c31v.", "c314.", "c31C.", "c316.", "c31E.", "c31w.", "c312.", "c31D.", "c317.", "c31F.", "c31y.", "l86..", "c51A.", "c51i.", "c51B.", "c51x.", "x0100", "x00zz", "c51v.", "c51w.", "c51C.", "c51D.", "c51F.", "c51E.", "c531."

Methylxanthine codes:

"c43..", "c431.", "c432.", "c433.", "c434.", "c435.", "c436.", "c437.", "c438.", "c439.", "c43A.", "c43B.", "c43a.", "c43b.", "c43c.", "c43d.", "c43e.", "c43f.", "c43g.", "c43h.", "c43i.", "c43j.", "c43k.", "c43m.", "c43n.", "c43o.", "c43p.", "c43q.", "c43r.", "c43s.", "c43t.", "c43u.", "c43v.", "c43w.", "c43x.", "c43y.", "c43z.", "c41..", "c411.", "c412.", "c413.", "c414.", "c415.", "c416.", "c417.", "c418.", "c419.", "c41A.", "c41B.", "c41C.", "c41a.", "c41b.", "c41c.", "c41d.", "c41e.", "c41f.", "c41g.", "c41h.", "c41i.", "c41j.", "c41k.", "c41m.", "c42..", "c42w.", "c421.", "c42x.", "c422.", "c42z.", "c424.", "c42y.", "c423.", "x01Ej", "x01Ek"

SABA+SAMA codes:

"c51A.", "c51B.", "c51C.", "c51D.", "c51E.", "c51F.", "c51G.", "c51H.", "c51v.", "c51w.", "c51x.", "c531.", "c51i.", "x0100", "x00zz"

ICS codes:

"c61..", "c611.", "c612.", "c613.", "c614.", "c615.", "c616.", "c617.", "c619.", "c61A.", "c61a.", "c61B.", "c61b.", "c61C.", "c61c.", "c61D.", "c61d.", "c61E.", "c61e.", "c61F.", "c61f.", "c61G.", "c61g.", "c61H.", "c61h.", "c61i.", "c61J.", "c61j.", "c61K.", "c61k.", "c61L.", "c61l.", "c61M.", "c61m.", "c61N.", "c61n.", "c61O.", "c61P.", "c61p.", "c61Q.", "c61q.", "c61R.", "c61r.", "c61S.", "c61s.", "c61T.", "c61t.", "c61U.", "c61u.", "c61V.", "c61v.", "c61W.", "c61w.", "c61X.", "c61x.", "c61Y.", "c61y.", "c61Z.", "c61z.", "c64..", "c641.", "c642.", "c643.", "c644.", "c645.", "c647.", "c648.", "c649.", "c64A.", "c64a.", "c64B.", "c64b.", "c64C.", "c64c.", "c64D.", "c64d.", "c64E.", "c64e.", "c64F.", "c64G.", "c64g.", "c64H.", "c64h.", "c64I.", "c64i.", "c64J.", "c64j.", "c64K.", "c64k.", "c64L.", "c64l.", "c64M.", "c64m.", "c64N.", "c64n.", "c64o.", "c64p.", "c64u.", "c64v.", "c64w.", "c64x.", "c64y.", "c64z.", "c65..", "c651.", "c652.", "c653.", "c654.", "c655.", "c656.", "c657.", "c658.", "c659.", "c65A.", "c65a.", "c65B.", "c65b.", "c65C.", "c65c.", "c65D.", "c65d.", "c65E.", "c65e.", "c65F.", "c65f.", "c65G.", "c65g.", "c65H.", "c65I.", "c65J.", "c65K.", "c65L.", "c65M.", "c65N.", "c65O.", "c65P.", "c65Q.", "c65R.", "c65S.", "c65T.", "c65U.", "c65V.", "c65W.", "c65X.", "c65Y.", "c65Z.", "c66..", "c661.", "c662.", "c663.", "c664.", "c665.", "c666.", "c667.", "c668.", "c669.", "c66A.", "c66a.", "c66B.", "c66b.", "c66C.", "c66c.", "c66D.", "c66d.", "c66E.", "c66e.", "c66F.", "c66f.", "c66G.", "c66g.", "c66H.", "c66h.", "c66I.", "c66J.", "c66K.", "c66L.", "c66M.", "c66N.", "c66P.", "c66Q.", "c66R.", "c66S.", "c66T.", "c66U.", "c66V.", "c66W.", "c66X.", "c66Y.", "c66Z.", "c681.", "c682.", "c683.", "c684.", "c691.", "c692.", "c69y.", "c69z.", "x00bU", "x00bV", "x00gE", "x00gF", "x00gG", "x00Hx", "x00Hy", "x00Hz", "x00I0", "x00I1", "x00I2", "x00I3", "x00I4", "x00QU", "x00QV", "x00z6", "x029g"

ICS+LABA codes:
"c67..", "c671.", "c672.", "c673.", "c674.", "c675.", "c67x.", "c67y.", "c67z.", "c6B..", "c6B1.", "c6B2.", "c6B3.", "c6B4.", "c1D..", "c1D1.", "c1D2.", "c1D3.", "c1D4.", "c1D5.", "c1D6.", "c1Du.", "c1Dv.", "c1Dw.", "c1Dx.", "c1Dy.", "c1Dz.", "c1c..", "c1c1.", "c1c2.", "c1c3.", "c1cx.", "c1cy.", "c1cz.", "c6A..", "c6A1.", "c6A2.", "c6Ay.", "c6Az."

LTRA codes:
"cA...", "cA1..", "cA11.", "cA12.", "cA13.", "cA14.", "cA15.", "cA16.", "cA1y.", "cA1z.", "cA2..", "cA21.", "cA22."

Omalizumab codes:
"ck1..", "ck11.", "ck12.", "ck13.", "ck14.", "ck15.", "ck16."

OCS codes:
"fe6..", "fe3..", "fe31.", "fe32.", "fe33.", "fe36.", "fe37.", "fe3A.", "fe3B.", "fe3C.", "fe3r.", "fe3s.", "fe3u.", "fe4..", "fe41.", "fe42.", "fe43.", "fe44.", "fe45.", "fe4e.", "fe4f.", "fe4g.", "fe4h.", "fe5..", "fe51.", "fe52.", "fe53.", "fe5f.", "fe5m.", "fe5n.", "fe5o.", "fe5p.", "fe61.", "fe62.", "fe64.", "fe65.", "fe66.", "fe67.", "fe68.", "fe69.", "fe6a.", "fe6c.", "fe6d.", "fe6e.", "fe6f.", "fe6g.", "fe6h.", "fe6i.", "fe6j.", "fe6k.", "fe6l.", "fe6m.", "fe6n.", "fe6o.", "fe6p.", "fe6q.", "fe6r.", "fe6s.", "fe6t.", "fe6v.", "fe6w.", "fe6z.", "fe7..", "fe71.", "fe72.", "fe73.", "fe74.", "fe75.", "fe76.", "fe77.", "fe78.", "fe79.", "fe7x.", "fe7y.", "fe7z.", "x00yP", "x01Mh", "x01Na", "x01Nb", "fe11.", "fe12.", "fe1x.", "fe1y.", "fe21.", "fe22.", "fe23.", "fe24.", "fe25.", "fe26.", "x01MW"

Asthma Exacerbation
"Xa1Hd", "Xafdz", "Xafdy", "Xafdj", "XacXk","XaINh", "38B8. ", "H333. ", "663y. "

Asthma Hospitalization
"663d"., "8H2P. "

Charlson Comorbidities

"G30y.", "G30..", "G307.", "G305.", "G300.", "G32..", "14AH.", "G302.", "G303.", "G301.", "G30yz", "G3011", "G30z.", "G3070", "G3071", "G30X0", "G600.", "G65z.", "G66..", "G660.", "G67y.", "Gyu66", "662M.", "F11x2", "G606.", "G61X0", "G62z.", "G669.", "G6774", "S621.", "70043", "G61..", "G618.", "G61X.", "G61X1", "G61z.", "G65y.", "G661.", "G68W.", "Gyu6.", "Gyu67", "S62..", "S628.", "G613.", "G63..", "G667.", "G671.", "G680.", "Gyu6D", "S627.", "G63z.", "G65..", "G65zz", "G666.", "G60z.", "G6410", "G663.", "G664.", "G665.", "G668.", "G671z", "Gyu65", "G633.", "G681.", "G6z..", "Gyu60", "S620.", "14A7.", "G602.", "G603.", "G604.", "G662.", "G6y..", "G6...", "G60..", "G601.", "G617.", "G63y.", "G67..", "G67z.", "Gyu61", "G605.", "G60X.", "G64..", "G68..", "Gyu62", "Gyu6F", "G623.", "G232.", "SP111", "G582.", "G58z.", "14A6.", "G5540", "G5800", "G580.", "G5810", "G5801", "G5802", "G581.", "G5803", "14AM.", "8B29.", "G58..", "662W.", "1O1..", "8H2S.", "8CL3.", "G5yA.", "H57y4", "N040A", "N040T", "Nyu45", "N0000", "N001.", "N0404", "N0406", "N040J", "N060.", "Nyu1G", "K01x4", "N040G", "N040H", "N04y0", "N04y1", "N20..", "F3964", "H57y1", "N04..", "N0407", "N040F", "N040P", "N2314", "N000.", "N0001", "N0405", "N041.", "N0010", "N2402", "N2z..", "Nyu10", "Nyu12", "F3712", "H572.", "N0004", "N000z", "N0012", "N040B", "N040D", "N040E", "N040M", "N2y..", "N0003", "N004.", "N0402", "N040C", "N200.", "2A42.", "N0403", "N040L", "N2400", "N240z", "F3961", "F3966", "N0002", "N040.", "N0408", "N0409", "N040K", "N0421", "N04X.", "N240.", "N2407", "Nyu11", "Nyu43", "H570.", "N047.", "E000.", "E0041", "Eu010", "Eu02z", "Eu02.", "Eu002", "Eu012", "E0010", "E041.", "Eu011", "Eu000", "Eu00z", "E004.", "E0042", "E004z", "Eu00.", "Eu02y", "E00..", "E001z", "Eu01z", "E0043", "E0040", "Eu001", "Eu01.", "Eu01y", "E001.", "Eu013", "Eu025", "C1000", "C103.", "C1074", "C109.", "C10y.", "C101.", "C1010", "C101z", "C10y1", "66AK.", "8A13.", "8H2J.", "C1030", "C103y", "C1073", "66AJ.", "C1001", "C1070", "C108.", "C1095", "C10A1", "C101y", "C1084", "C1093", "C1094", "C10yz", "C10z1", "C10zz", "G73y0", "L1806", "66A5.", "C1099", "66AJz", "C1020", "C107.", "C1088", "C10A0", "Cyu20", "C10..", "C1071", "C10A.", "C10B0", "C10zy", "Cyu2.", "66AI.", "C100z", "C1072", "C10z.", "L180X", "C1011", "C102.", "C107z", "66AS.", "C100.", "C1021", "C102z", "C103z", "C1085", "C1086", "C1097", "C10yy", "L1805", "C108E", "C1089", "C109F", "C109D", "C109J", "C109G", "66AV.", "C108G", "C10D.", "C109K", "C10E.", "C10F.", "C10FJ", "C10F9", "C10FM", "C10EM", "C10EE", "C10EN", "C10FN", "C10FL", "C10E8", "C10FF", "C10E9", "C10F7", "C10E4", "C10F4", "C10EL", "C10F5", "8BL2.", "C10E5", "C10EK", "C10FP", "C10FD", "C10G.", "C10FG", "C10E6", "C10M.", "C10N.", "C10EA", "C10H.", "C10EG", "J61..", "J615C", "C3104", "J614z", "J615z", "J6161", "C3500", "J6141", "J6151", "J6160", "J614.", "J615F", "J614y", "J6153", "J615y", "J6002", "J615.", "J615B", "J616z", "J6012", "J6143", "J6170", "J6140", "J6157", "J616.", "J633.", "J6356", "J612.", "J6142", "J6150", "J6154", "J615A", "Jyu71", "J61y3", "J11..", "J110y", "J11yy", "J1201", "J1214", "J121y", "J12y4", "J12z.", "J140z", "ZV12C", "761Jy", "J1102", "J1114", "J13y.", "J13yy", "J14..", "J1412", "761J1", "J1111", "J111z", "J120.", "J120y", "J1301", "J1303", "J13z.", "J1400", "J14y3", "76121", "761J0", "J1020", "J1100", "J11y3", "J122.", "J12y0", "J12yy", "J130.", "J130z", "J131y", "J140.", "J1402", "J14y.", "ZV127", "J1101", "J111y", "J11z.", "J12yz", "J1300", "J131.", "J1313", "J140y", "J110.", "J1112", "J1203", "J124.", "J130y", "J1314", "J13y0", "J13y2", "J13y4", "J1413", "761Jz", "J11y.", "J12..", "J1202", "J1204", "J1302", "J1304", "J13y3", "J1403", "J14y4", "761J.", "J11y4", "J1200", "J121.", "J1211", "J123.", "J12y.", "J12y2", "J1312", "J1404", "J14y1", "J14yy", "J1110", "J1113", "J11y0", "J13..", "J1310", "J13yz", "J141.", "J1411", "J14y2", "J14yz", "76125", "76270", "J1104", "J110z", "J111.", "J11y1", "J120z", "J1210", "J1414", "J141z", "J1103", "J11y2", "J11yz", "J1213", "J121z", "J12y3", "J1311", "J131z", "J13y1", "J1401", "J1410", "J141y", "J14y0", "J14z.", "761D6", "J112.", "7A134", "G713.", "G7130", "G7324", "R0543", "7A13.", "G7150", "G715.", "G73..", "G7323", "G73y.", "Gyu71", "R0542", "R054z", "14AE.", "C107.", "G712.", "G7141", "G7311", "G711.", "Gyu72", "G710.", "G714.", "G73z0", "Gyu74", "7A14.", "R054.", "7A113", "G71..", "G7140", "G718.", "G71z.", "G7321", "G73yz", "G7322", "7A112", "7A144", "G7160", "G73z.", "G73zz", "14NB.", "2I16.", "G716.", "G732.", "G7320", "R0540", "663N1", "663f.", "9OJ1.", "H300.", "H31..", "H3120", "H341.", "H354.", "H41z.", "H420.", "663N.", "663N0", "663P.", "663W.", "H32y2", "H331.", "H33z.", "H356.", "H35y6", "H35z.", "H35zz", "H42..", "H441.", "H47y0", "H4z..", "H581.", "663h.", "9OJA.", "H30z.", "H3101", "H311z", "H31yz", "H3200", "H3202", "H32y1", "H3310", "H33zz", "H340.", "H34z.", "H35y.", "H35y5", "H43z.", "H460z", "Hyu30", "SK07.", "H32..", "H32yz", "H32z.",

"H330.", "H33z0", "H41..", "14B4.", "663e.", "H30..", "H33..", "H3311", "H35y7", "H435.", "H4640", "H57yz", "H582.", "H3110", "H312z", "H33z2", "H34..", "H352.", "H353.", "H430.", "H432.", "H440.", "H442.", "H3100", "H3121", "H313.", "H31y1", "H31z.", "H331z", "H350.", "H351.", "H352z", "H35z1", "H3z..", "H40..", "H460.", "Hyu43", "173A.", "663V0", "H3111", "H312.", "H321.", "H3300", "H330z", "H332.", "H43..", "H4642", "663V1", "663V2", "H310.", "H311.", "H320.", "H320z", "H32y.", "H32y0", "H3301", "H35y3", "H423.", "H4641", "H4y10", "8H2P.", "H310z", "H333.", "H33z1", "H3520", "H355.", "H35yz", "H410.", "H45..", "H57y.", "663V3", "H31y.", "H322.", "H35..", "H3521", "H42z.", "H431.", "H434.", "Hyu40", "Hyu41", "663v.", "663q.", "663r.", "66YC.", "663s.", "663t.", "663u.", "1O2..", "66YP.", "H334.", "173c.", "663N1", "663f.", "9OJ1.", "H3120", "663N.", "663N0", "663P.", "663W.", "H331.", "H33z.", "H47y0", "663h.", "9OJA.", "H3310", "H33zz", "H330.", "H33z0", "14B4.", "663e.", "H33..", "H3311", "H35y7", "H33z2", "H331z", "173A.", "663V0", "H3300", "H330z", "H332.", "663V1", "663V2", "H3301", "8H2P.", "H333.", "H33z1", "663V3", "663v.", "663q.", "663r.", "66YC.", "663s.", "663t.", "663u.", "1O2..", "66YP.", "H334.", "173c.", "B0001", "B002.", "B0022", "B0033", "B010.", "B010z", "B023.", "B060.", "B0622", "B0623", "B0710", "B0720", "B07z.", "B08..", "B082.", "B0zy.", "B105.", "B110z", "B112.", "B120.", "B140.", "B15..", "B150z", "B1612", "B161z", "B172.", "B18..", "B1801", "B18y.", "B18y0", "B18y2", "B2011", "B2012", "B203.", "B204.", "B205.", "B20z.", "B2133", "B23z.", "B26..", "B30..", "B302z", "B3055", "B3133", "B3151", "B3152", "B3153", "B323.", "B3232", "B324z", "B326z", "B3276", "B3320", "B3332", "B335.", "B3350", "B3351", "B3352", "B3356", "B336.", "B3377", "B342.", "B34yz", "B35..", "B3500", "B410.", "B43..", "B431z", "B43y.", "B45y0", "B47z.", "B480.", "B490.", "B4Az.", "B505.", "B510z", "B5150", "B5200", "B5210", "B5211", "B5230", "B5246", "B540.", "B5504", "B550z", "B5512", "B553z", "B55y0", "B55y1", "B6003", "B6006", "B600z", "B6012", "B613z", "B6140", "B6157", "B6162", "B6208", "B6216", "B6223", "B6227", "B6238", "B6243", "B6274", "B62x3", "B62x4", "B62y6", "B63..", "B630.", "B65..", "B653.", "B662.", "B675.", "B690.", "B692.", "Byu23", "Byu33", "Byu51", "ByuA2", "ByuDF", "ZV100", "ZV102", "ZV107", "B0023", "B0130", "B0131", "B01z.", "B021.", "B050.", "B0513", "B055.", "B064z", "B0731", "B103.", "B10z.", "B1100", "B111.", "B113.", "B114.", "B115.", "B16..", "B1610", "B180z", "B18y5", "B210.", "B213.", "B2130", "B2131", "B2211", "B2230", "B2241", "B22z.", "B23..", "B2414", "B24X.", "B2z..", "B3006", "B3020", "B3030", "B3032", "B305.", "B3050", "B3064", "B306z", "B3071", "B3085", "B3100", "B3120", "B3121", "B3132", "B314.", "B315z", "B3221", "B3231", "B325.", "B325z", "B3271", "B3273", "B32y0", "B330.", "B3310", "B3321", "B3333", "B3355", "B3358", "B3372", "B341.", "B345.", "B350.", "B3501", "B350z", "B3y..", "B4100", "B420.", "B44z.", "B4500", "B451.", "B451z", "B45z.", "B49..", "B49y.", "B4A10", "B4Ay.", "B500.", "B5100", "B511.", "B512.", "B512z", "B51y.", "B521z", "B524X", "B543.", "B545z", "B5500", "B554.", "B55yz", "B6...", "B600.", "B6101", "B611.", "B6111", "B6116", "B6122", "B6143", "B6154", "B61z.", "B6210", "B6211", "B6214", "B624z", "B62y1", "B62z6", "B62zz", "B631.", "B64y.", "B64y0", "B64y1", "B64yz", "B653z", "B66..", "B67..", "By...", "Byu2.", "Byu21", "Byu3.", "Byu5.", "Byu58", "Byu82", "Byu9.", "ByuD2", "ByuD6", "ByuDC", "Bz...", "ZV104", "ZV10y", "B....", "B000.", "B0040", "B00z1", "B013z", "B016.", "B02..", "B041.", "B05z0", "B0600", "B07..", "B073z", "B07y.", "B11y0", "B133.", "B136.", "B14y.", "B1500", "B1502", "B1510", "B1613", "B170.", "B180.", "B1z10", "B1zz.", "B2000", "B21..", "B2200", "B220z", "B221z", "B222.", "B224.", "B22y.", "B3000", "B302.", "B3021", "B3033", "B3054", "B3058", "B3063", "B307z", "B308.", "B3088", "B3103", "B3104", "B3110", "B3124", "B3125", "B314z", "B315.", "B322z", "B3241", "B3255", "B33..", "B3312", "B333.", "B3330", "B334.", "B337.", "B3371", "B3401", "B344.", "B347.", "B34y0", "B41y.", "B41y0", "B41y1", "B41z.", "B4302", "B431.", "B441.", "B4702", "B48z.", "B493.", "B49y0", "B4A0.", "B515.", "B515z", "B5173", "B5202", "B524.", "B5241", "B524W", "B52y.", "B541.", "B542z", "B5531", "B55y.", "B6001", "B6008", "B6015", "B6016", "B6025", "B6105", "B610z", "B6120", "B6163", "B6167", "B6168", "B61z5", "B61zz", "B620.", "B6200", "B6217", "B621z", "B6235", "B624.", "B627D", "B6303", "B64..", "B64z.", "B67z.", "B68..", "B680.", "B69..", "Byu32", "Byu70", "ByuD8", "ZV106", "B0031", "B003z", "B004.", "B0043", "B00zz", "B0110", "B013.", "B014.", "B064.", "B0641", "B06y.", "B06z.", "B071.", "B0730", "B080.", "B081.", "B0z..", "B102.", "B110.", "B12..", "B12y.", "B141.", "B1503", "B163.", "B16y.", "B175.", "B17y.", "B181.", "B18y4", "B18y6", "B1z..", "B1z11", "B200.", "B202.", "B212.", "B225.", "B2410", "B2411", "B303.", "B304.", "B3041", "B3042", "B3057",

"B3061", "B308z", "B3111", "B3115", "B3220", "B3234", "B3251", "B3254", "B3256", "B3262", "B32y.", "B3354", "B335A", "B3370", "B3378", "B337z", "B33X.", "B34z.", "B40..", "B41yz", "B440.", "B45..", "B46..", "B49z.", "B4Ay0", "B5003", "B501.", "B5121", "B521.", "B545.", "B5451", "B5503", "B55y2", "B55z.", "B6004", "B6100", "B6102", "B6123", "B6127", "B6128", "B613.", "B6132", "B62..", "B6203", "B6234", "B623z", "B6241", "B6244", "B6245", "B6246", "B6262", "B6265", "B627X" , "B62x5", "B62y2", "B62y3", "B62z0", "B62z1", "B651.", "B6512", "B6531", "B66y.", "B66y0", "B691.", "B6y1.", "Byu10", "Byu25", "Byu50", "Byu54", "Byu5A", "ByuD.", "ZV105", "B001.", "B00z0", "B012.", "B03z.", "B052.", "B060z", "B0621", "B071z", "B072z", "B101.", "B1101", "B116.", "B13z.", "B142.", "B1420", "B16z.", "B173.", "B18y1", "B18y7", "B1z1z", "B2001", "B201.", "B2010", "B21y.", "B2201", "B223.", "B232.", "B240.", "B241z", "B242.", "B3...", "B3005", "B3051", "B3056", "B305A", "B305z", "B3072", "B3083", "B31..", "B311z", "B3140", "B3141", "B31z.", "B3272", "B3341", "B3357", "B3364", "B3365", "B336z", "B3373", "B4...", "B41..", "B411.", "B412.", "B4700", "B4710", "B4711", "B48..", "B481.", "B491.", "B4A1.", "B4y..", "B5000", "B510.", "B5151", "B516.", "B5231", "B523z", "B5244", "B54..", "B5421", "B544.", "B54X.", "B54y.", "B6018", "B6026", "B6130", "B6138", "B6145", "B616.", "B61z1", "B61z6", "B622z", "B6242", "B6256", "B6260", "B6268", "B6270", "B6272", "B627C", "B62x2", "B62z2", "B6302", "B641.", "B6510", "B65z.", "B68y.", "Byu1.", "Byu11", "Byu13", "Byu24", "Byu31", "Byu53", "Byu73", "Byu8.", "ByuA0", "ByuD4", "ByuDE", "ByuE.", "ZV101", "B0000", "B0042", "B005.", "B006.", "B011z", "B015.", "B040.", "B042.", "B05y.", "B062z", "B065.", "B1110", "B11y1", "B11yz", "B123.", "B124.", "B137.", "B14..", "B143.", "B150.", "B15z.", "B160.", "B174.", "B17y0", "B17z.", "B18y3", "B18yz", "B1z2.", "B2...", "B2221", "B231.", "B301.", "B3035", "B3081", "B3084", "B3086", "B308C", "B308D", "B3105", "B310z", "B3112","B313.", "B316.", "B317.", "B3230", "B3240", "B3257", "B3260", "B3278", "B3279", "B327z", "B3334", "B334z", "B3353", "B3360", "B3379", "B339.", "B33y.", "B33z0", "B35z0", "B3z..", "B430.", "B453.", "B470.", "B483.", "B48yz", "B4A..", "B5011", "B5104", "B5105", "B513.", "B514.", "B51y0", "B52..", "B523.", "B52z.", "B5505", "B602.", "B6021", "B6022", "B610.", "B6114", "B6133", "B615z", "B616z", "B61z4", "B6215", "B6231", "B6236", "B6251", "B6252", "B6253", "B626.", "B6271", "B6275", "B6276", "B6277", "B62y.", "B62z5", "B6530", "B67y.", "B67y0", "B681.", "B68z.", "B6z..", "Byu0.", "Byu22", "Byu40", "ByuD3", "ByuD7", "ByuD9", "B00..", "B0010", "B0011", "B004z", "B02y.", "B03y.", "B056.", "B0601", "B0620", "B0711", "B072.", "B0721", "B0732", "B0z1.", "B1...", "B10y.", "B11..", "B13y.", "B161.", "B171.", "B18z.", "B2002", "B222z", "B2z0.", "B2zz.", "B300.", "B3002", "B300C", "B3031", "B3034", "B3044", "B304z", "B3053", "B3089", "B30X.", "B3102", "B3123", "B313z", "B3235", "B3252", "B326.", "B3263", "B327.", "B3335", "B3361", "B3374", "B340.", "B3400", "B340z", "B343.", "B35z.", "B410z", "B4303", "B44..", "B444.", "B450.", "B452.", "B485.", "B487.", "B495.", "B4A2.", "B4A4.", "B500z", "B501z", "B504.", "B506.", "B5103", "B5245", "B5511", "B553.", "B5532", "B6000", "B601.", "B6028", "B602z", "B60y.", "B6108", "B6112", "B6115", "B6125", "B6134", "B6142", "B6146", "B6204", "B6205", "B6206", "B6213", "B6220", "B6221", "B6226", "B6247", "B6250", "B625z", "B6264", "B62x6", "B62y8", "B62z.", "B62z3", "B6301", "B642.", "B661.", "B671.", "B673.", "B682.", "B6y0.", "Byu..", "Byu41", "Byu72", "Byu80", "ByuD0", "ByuDA", "B001z", "B0021", "B003.", "B00y.", "B0111", "B0511", "B061.", "B063.", "B067.", "B0z2.", "B106.", "B11z.", "B121.", "B130.", "B14z.", "B1511", "B1512", "B1513", "B1611", "B17yz", "B1800", "B182.", "B1z0.", "B20..", "B2003", "B200z", "B206.", "B211.", "B2132", "B22..", "B3001", "B3003", "B3008", "B3022", "B303z", "B3062", "B307.", "B311.", "B3113", "B31y.", "B322.", "B3270", "B3274", "B32z.", "B3331", "B3375", "B346.", "B4300", "B4701", "B470z", "B482.", "B484.", "B48y1", "B48y2", "B4A11", "B4z..", "B5...", "B50..", "B5010", "B507.", "B5070", "B5071", "B51yz", "B5232", "B5401", "B54z.", "B550.", "B5502", "B551z", "B552.", "B6005", "B6027", "B6107", "B6110", "B6117", "B612.", "B6136", "B6137", "B6141", "B6151", "B6152", "B6156", "B6161", "B6166", "B6201", "B6202", "B622.", "B6222", "B6232", "B625.", "B6263", "B6266", "B6267", "B627.", "B627W", "B62x.", "B62xX", "B62y7", "B62yz", "B62z8", "B672.", "B6z0.", "Byu55", "Byu6.", "Byu7.", "ByuA.", "ByuD1", "ByuE0", "ZV10.", "ZV10z", "B002z", "B0032", "B0041", "B00z.", "B01..", "B017.", "B02z.", "B030.", "B051z", "B054.", "B0550", "B0551", "B057.", "B0602", "B0640", "B066.", "B06yz", "B0z0.", "B10..", "B104.", "B111z", "B117.", "B134.", "B1501", "B151z", "B1z1.", "B1zy.", "B214.", "B21z.", "B220.", "B221.", "B23y.", "B2413", "B2zy.", "B300z", "B3040", "B3059", "B306.", "B3060", "B309.", "B30W.",

"B30z.", "B3114", "B3122", "B3131", "B31z0", "B320.", "B3253", "B3275", "B3277", "B331.", "B3311",
"B3322", "B333z", "B3362", "B4101", "B4301", "B4310", "B442.", "B4501", "B4510", "B454.", "B471.",
"B48y0", "B497.", "B4A1z", "B5001", "B507z", "B5102", "B517.", "B5170", "B51z.", "B5212", "B522.",
"B52W.", "B540z", "B5452", "B551.", "B5510", "B60..", "B6007", "B6011", "B6024", "B6103", "B6104",
"B612z", "B6135", "B614.", "B6155", "B6160", "B6164", "B61z7", "B6207", "B6228", "B6233",
"B6237", "B6248", "B6254", "B6255", "B6258", "B6261", "B6278", "B62y4", "B62y5", "B62z7",
"B6300", "B63y.", "B652.", "B660.", "B66z.", "B670.", "Byu4.", "Byu57", "Byu71", "Byu81", "Byu90",
"B0...", "B000z", "B0020", "B01y.", "B022.", "B03..", "B05..", "B051.", "B0510", "B055z", "B05z.",
"B062.", "B06y0", "B073.", "B084.", "B11y.", "B12z.", "B13..", "B131.", "B138.", "B151.", "B152.",
"B162.", "B2013", "B201z", "B2220", "B2231", "B223z", "B226.", "B230.", "B241.", "B24y.", "B24z.",
"B3004", "B3009", "B300A", "B3043", "B305D", "B3070", "B3082", "B308A", "B310.", "B32..", "B324.",
"B3250", "B3265", "B332.", "B3340", "B3359", "B335z", "B33z.", "B42..", "B430z", "B43z.", "B450z",
"B45y.", "B47..", "B4703", "B492.", "B4A00", "B4A3.", "B508.", "B50y.", "B5171", "B51y1", "B51y2",
"B5201", "B5240", "B53..", "B5400", "B5420", "B5530", "B555.", "B6002", "B6010", "B601z", "B6020",
"B6106", "B6124", "B6144", "B6148", "B615.", "B6150", "B6153", "B61z0", "B61z2", "B621.", "B6212",
"B6230", "B6257", "B626z", "B6273", "B62x1", "B62y0", "B62z4", "B63z.", "B64y2", "B65y1", "B674.",
"B67yz", "Byu12", "Byu30", "Byu52", "Byu59", "ByuA1", "ByuA3", "ByuDD", "ZV103", "B0030",
"B007.", "B0100", "B011.", "B020.", "B031.", "B04..", "B04y.", "B04z.", "B0512", "B053.", "B06..",
"B070.", "B074.", "B083.", "B08y.", "B08z.", "B0zz.", "B100.", "B1111", "B122.", "B132.", "B135.",
"B1514", "B17..", "B1802", "B20y.", "B213z", "B215.", "B2210", "B2240", "B224z", "B24..", "B2412",
"B243.", "B25..", "B3007", "B3052", "B305C", "B3080", "B3087", "B3101", "B312.", "B3126", "B312z",
"B3130", "B3150", "B321.", "B3233", "B323z", "B3258", "B3261", "B3264", "B332z", "B3363",
"B3376", "B34..", "B34y.", "B35zz", "B432.", "B443.", "B44y.", "B45X.", "B486.", "B48y.", "B494.",
"B496.", "B5002", "B502.", "B503.", "B50z.", "B51..", "B5101", "B5120", "B5172", "B517z", "B520.",
"B520z", "B5242", "B5243", "B525.", "B52X.", "B542.", "B5450", "B55..", "B5501", "B59zX", "B6013",
"B6014", "B6017", "B6023", "B60z.", "B61..", "B6113", "B6118", "B611z", "B6121", "B6126", "B6131",
"B6147", "B614z", "B6158", "B6165", "B61z3", "B61z8", "B620z", "B6218", "B6224", "B6225", "B623.",
"B6240", "B62x0", "B640.", "B650.", "B651z", "B65y.", "B65y0", "B65yz", "B66yz", "B6y..", "Byu20",
"Byu42", "Byu43", "Byu56", "ByuC0", "ByuD5", "B30z0", "B3065", "C104.", "C1080", "C108D",
"C1092", "C1096", "F4640", "K01x1", "C106.", "C109C", "C1040", "C104y", "C106z", "C1051", "F372.",
"F4203", "C1081", "C1087", "C1090", "F420z", "C1082", "F3813", "F4202", "C104z", "C108B", "F374z",
"F4201", "C109B", "C109A", "F420.", "F4204", "C1050", "C105y", "C105z", "C1091", "F3y0.", "C105.",
"C1061", "C106y", "C108C", "C109E", "C108F", "C108H", "C109H", "2BBL.", "F4206", "F4208", "C108J",
"C10FC", "C10FH", "C10ED", "C10F6", "C10FA", "C10E0", "C10E1", "C10F2", "C10FE", "C10F0",
"F4207", "C10EC", "C10FB", "2BBP.", "2BBQ.", "2BBR.", "2BBS.", "2BBV.", "C10E2", "C10F1", "C10EF",
"2BBk.", "C10EB", "C10EQ", "C10FR", "F222.", "F241.", "F2300", "F221.", "F2411", "F2410", "F230.",
"F223.", "F220.", "F141.", "F22..", "F22z.", "K001.", "K032.", "K080.", "K0A32", "K02yz", "K03..",
"K034.", "K05..", "K0A33", "K0A55", "K041.", "K021.", "K032y", "K042.", "K0800", "K08yz", "K1010",
"K0...", "K012.", "K02..", "K023.", "K02z.", "K0801", "Kyu2.", "K02y0", "K032z", "K035.", "K0A34",
"K0A37", "K0803", "K1001", "K1011", "Kyu20", "K080z", "K08z.", "Kyu21", "K060.", "K081.", "K019.",
"K022.", "K02y2", "K02y3", "K050.", "K06..", "K08y0", "K0A35", "K1000", "14D1.", "K0320", "K04y.",
"K04z.", "K0802", "1Z10.", "1Z12.", "1Z13.", "1Z14.", "1Z11.", "B560.", "B5612", "B5614", "B561z",
"B5620", "B5633", "B572.", "B573.", "B575z", "B58..", "B583z", "B58y3", "B58y8", "ByuC1", "B56..",
"B5608", "B5610", "B5642", "B570.", "B574.", "B575.", "B576z", "B5821", "B582z", "B58y.", "B58y4",
"B590.", "B594.", "B5y..", "B562.", "B5632", "B564.", "B565.", "B57..", "B5751", "B5812", "B5824",
"B5831", "B585.", "B586.", "B58y2", "B59..", "B5618", "B5621", "B5624", "B563z", "B564z", "B5654",
"B56y.", "B5740", "B5761", "B5825", "B584.", "B587.", "B5z..", "ByuC.", "ByuC5", "B5600", "B5604",
"B5616", "B5617", "B562z", "B577.", "B5811", "B58y5", "B58y9", "B591.", "B59z.", "ByuC4", "B5603",
"B5605", "B5623", "B5631", "B5641", "B5653", "B56z.", "B571.", "B5762", "B57y.", "B57z.", "B580.",
"B581z", "B593.", "ByuC2", "ByuC7", "B153.", "B5606", "B5609", "B5613", "B563.", "B5650", "B5741",
"B5742", "B5810", "B5822", "B58y0", "B58y7", "ByuC8", "B574z", "B581.", "B5830", "B58y1", "B592X",

"B560z", "B5619", "B5652", "B565z", "B576.", "B5820", "B5826", "B5832", "B58yz", "B5607", "B561.", "B5615", "B5750", "B5823", "B583.", "B58z.", "ByuC3", "B5601", "B5602", "B5611", "B5622", "B5630", "B5640", "B5651", "B5760", "B582.", "B58y6", "B592.", "ByuC6", "B5850", "G85..", "G850.", "J62y.", "A704z", "G851.", "G8522", "J624.", "J62z.", "G8521", "Gyu94", "760F3", "J623.", "J622.", "G8523", "G852z", "G852.", "G8520", "G858.", "A7898", "A788.", "A7885", "A788X", "A7892", "A7895", "AyuC1", "AyuC7", "A7881", "A7893", "A7896", "AyuC2", "AyuCA", "A788U", "A789.", "AyuC0", "AyuC8", "A788y", "A7894", "AyuC5", "AyuC9", "AyuCC", "A788W", "A789X", "AyuC3", "AyuC4", "AyuC6", "A7886", "A788z", "A789A", "A7882", "A7883", "A7884", "A788V", "A7891", "AyuC.", "AyuCD", "A7890", "A7899", "AyuCB"

Age

Age was calculated using date of birth rather than read codes

Height

"229.."

Weight

"22A.."

Smoking

Current smoking:

"137..", "1372.", "1373.", "1374.", "1375.", "1376.", "137a.", "137b.", "137c.", "137C.", "137d.", "137D.", "137e.", "137f.", "137G.", "137h.", "137H.", "137J.", "137m.", "137M.", "137o.", "137P.", "137Q.", "137R.", "137V.", "137X.", "137Y.", "137Z.", "Ub1tl", "Ub1tJ", "Ub1tK", "Ub1tR", "Ub1tS", "Ub1tT", "Ub1tU", "Ub1tV", "Ub1tW", "XaBSp", "Xallu", "XalkW", "XalkY", "Xaltg", "XaJX2", "XaLQh", "XaWNE", "XaZIE", "XE0og", "XE0oi", "XE0oq", "XE0or"

Ex-smoking:

"1377.", "1378.", "1379.", "137A.", "137B.", "137F.", "137j.", "137K.", "137l.", "137N.", "137O.", "137S.", "137T.", "Ub0p1", "Ub1na", "Xa1bv", "XaQ8V", "XE0oj", "XE0ok", "XE0ol", "XE0om", "XE0on", "XE0op", "137K0", "XaQzw"

Passive smoking:

"13WF.", "XM1Jh", "13WF4", "137I.", "137I0", "Ub0pe", "Ub0pf", "Ub0pg"

Non-smoking:

"1371.", "XE0oh", "137U.", "XaFvq"
